# Understanding Animal Detection of Precursor Earthquake Sounds

**DOI:** 10.3390/ani7090066

**Published:** 2017-08-31

**Authors:** Michael Garstang, Michael C. Kelley

**Affiliations:** 1 Department of Environmental Sciences, University of Virginia, Charlottesville, VA 22904, USA; 2 School of Electrical and Computer Engineering, Cornell University, Ithaca, NY 14853, USA; mck13@cornell.edu

**Keywords:** infrasound, electrophonics, sound detection, animal behavior, earthquake prediction

## Abstract

**Simple Summary:**

Scientists and amateur seismologists, particularly in China and Japan, have attempted over hundreds of years to use the behavior of both wild and domestic animals, disturbed by some sensory input well before a major earthquake, as a predictor of that event. The most striking case occurred in 1975, when, in addition to other precursor events, domestic and wild animals in and around the city of Haicheng, China behaved in an extremely anomalous fashion. The city was partially evacuated and many thousands of lives were saved. Re-analysis of these data, however, found it difficult to reconstruct the source, timing and impact of the actual earthquake warnings. Here we provide, for the first time, an explanation of how animals might detect, in advance, the occurrence of an earthquake and why inconsistencies are likely in such a prediction.

**Abstract:**

We use recent research to provide an explanation of how animals might detect earthquakes before they occur. While the intrinsic value of such warnings is immense, we show that the complexity of the process may result in inconsistent responses of animals to the possible precursor signal. Using the results of our research, we describe a logical but complex sequence of geophysical events triggered by precursor earthquake crustal movements that ultimately result in a sound signal detectable by animals. The sound heard by animals occurs only when metal or other surfaces (glass) respond to vibrations produced by electric currents induced by distortions of the earth’s electric fields caused by the crustal movements. A combination of existing measurement systems combined with more careful monitoring of animal response could nevertheless be of value, particularly in remote locations.

## 1. Introduction

Attempts to use the behavior of wild and domestic animals, disturbed by some sensory input, to predict the occurrence of an earthquake, have a long history. Despite the many occasions when extreme adherent animal behavior has been observed, no consistent prediction of an earthquake, based upon animal behavior, has been reported [1,2].

No predictions of earthquakes have withstood careful scrutiny [3,4]. The magnitude 7.3, 4 February 1975, Haicheng, China, earthquake was considered to be the only major earthquake ever to have been predicted. Wang et al. [3], upon exhaustive reanalysis, found it difficult to determine the source, timing and impact of the actual earthquake warnings issued.

In this paper, we present evidence that animals are capable of hearing a wide range of sounds, with frequencies ranging from ultra- to infrasound (kHz-Hz) and from biotic to abiotic sources. The sounds dealt with in this study are abiotic sounds not commonly considered within the biological acoustic literature [5,6].

We call upon our recent discoveries which describe sound signals, detectable by animals, that might serve as earthquake predictors [7,8]. Because of the use of sound made by elephants and the extensive literature documenting this behavior, we use elephants as the most appropriate vehicle to relate animal detection of sounds to events associated with, earthquakes. These deductions can then be credibly transferred to other animals, both domestic and wild, potentially detecting earthquakes. We acknowledge that our focus is upon the detection of sound by animals and not upon their response to other senses such as smell, taste and touch.

Finally, we will evaluate these findings and address their contribution to earthquake prediction and the potential role that can be played by documenting animal response.

## 2. Previous Research

### 2.1. Animal Use of Sound

Animals generate and detect sounds over a wide range of frequencies and for many purposes. Bats, for example, use ultra sound (20–200 kHz) to navigate and capture prey [9]. Elephants use infrasound (<20 Hz) for long-range communication which is vital to their reproduction, resource utilization and predator avoidance [5]. Communication has been widely studied among the higher order mammals, including the primates and elephants [5,10,11].

Far fewer studies have investigated animal use of abiotic sounds, such as thunder produced by rainstorms or noise generated prior to and during earthquakes. Kelley and Garstang [12] have reported the elephant’s ability to detect thunderstorms. Garstang has done extensive work on the use of infrasound by elephants [12,13,14,15,16]. Garstang has also suggested that elephants up to 1000 km away from the epicenter detected the breaking of the tsunami on the shores of Sumatra [17].

Kelley and Garstang [12] estimated that the acoustic pressure generated by a thunderstorm at a distance of 10 km from the receiver is at least 6 × 10^−3^ Pa. With the threshold of hearing of humans at a frequency of 1 kHz at 2 × 10^−5^ Pa, humans can hear these sounds [17,18]. Elephants and most other animals are capable of hearing sounds much lower than 2 × 10^−5^ Pa. These animals are also able to hear such sounds at much greater distances, as described below.

The Boxing Day Tsunami on 26 December 2004 was created by a magnitude 9.0 earthquake, 160 km off the northwest coast of Sumatra, one minute before 0800 local time (00:58:53 UCT). The earthquake resulted in a 15.6 m (50 ft) wave that crashed onto the coast of Sumatra. This wave impacted the shoreline, producing low-frequency sounds at and below 100 Hz with much of the energy residing in infrasonic frequencies between 1 Hz and 10 Hz. These sounds travel in the atmosphere at the speed of sound. At the tropical ocean surface this speed will be about 1260 km/h. The tsunami, in contrast, travels at a speed of 700 km/h. Elephants at locations 1000 km distant from Sumatra (Phuket, Thailand and Yala National Park, Sri Lanka) detected and responded to this sound wave 38.1 min prior to the arrival of the tsunami. No elephant in either location was lost or injured. In contrast, a significant number of people on the beaches of Thailand lost their lives [17].

Kelley and Price [8], argue that large electric fields are generated in the wake of a meteor. These electric fields can produce a current which, in turn, produces radio waves and generate sound which is reportedly heard by humans when observing meteors. These sound waves can cause metal surfaces, glasses and other objects to vibrate producing audible sounds at frequencies between 20 Hz and 20,000 Hz. The coupling between radiowaves and sound waves is called electrophonics and is well established. As noted in many references reported in Kelley and Price [8], and elsewhere, humans can hear audio signals at the same time they see the meteors. Such a transition from electric fields to sound waves would, to a far greater extent, be detectable to many other animal species. We draw upon this theoretical evidence to demonstrate that earthquake precursor signals can generate such sounds and that these sounds are detectable by animals.

### 2.2. Precursor Earthquake Signals

Kelley et al. [7] show that ionospheric signatures of the Slant Total Electron Content (STEC) record fluctuations that are induced by earthquake precursor crustal movements. These signals are transmitted at nearly the speed of light and guided by the earth’s magnetic field to the ionosphere.

The Slant Total Electron Content is measured by using two frequencies transmitted from the Global Positioning Signals (GPS) satellites to the earth. The time difference between these two signals is directly proportional to the number of electrons between the satellites and the ground. This time-delay gives a direct estimate of changes in the electron content of the ionosphere. The satellites are at an orbital height of approximately 40,000 km, well above the ionosphere.

Changes in the line-of-sight electron content are triggered by electric fields generated by failure in the earth’s strata preceding an earthquake [7]. These changes are detected and recorded by ground-based GPS recorded at GPS stations.

Figure 1 from Kelley et al. [7], show the STEC changes in the form of enhancements (red) and depressions (blue) over a range of latitudes from 15 Japanese ground GPS stations created by an earthquake. The time of the earthquake is marked by the central black vertical line.

**Figure 1:**
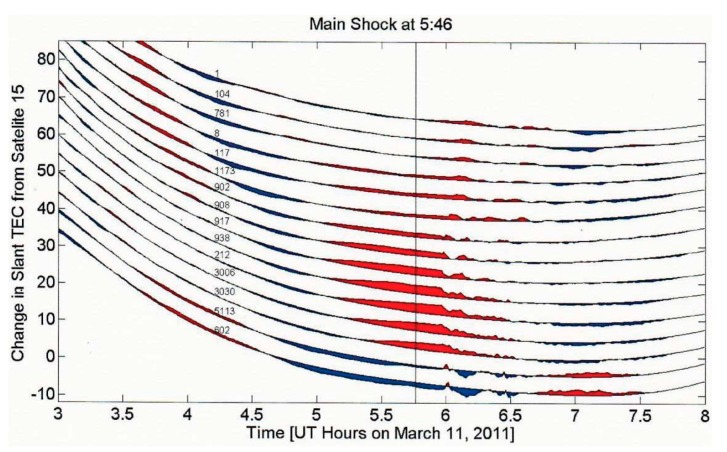
Total Electron Context (STEC) time series from fifteen ground stations and Satellite 15. The ground station numbers are listed at the left and time of the quake is indicated by the vertical line. Red fills correspond to STEC enhancements and blue fills to STEC depressions (After Kelley et al. 2017 [7], reproduced with permission of the American Geophysical Union).

**Figure 2:**
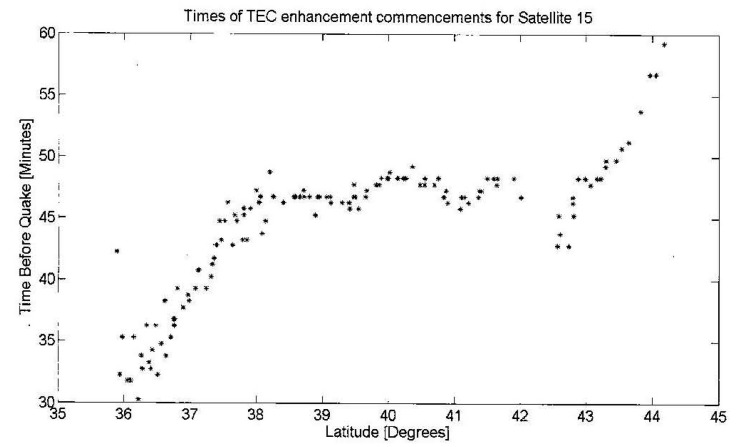
Time intervals between STEC enhancement commencements for latitudes between 38° and 43°. The simultaneously of the STEC enhancements show that they are tied to the earth and not due to a wave propagating in the atmosphere. The sloping points at latitudes below 38° and higher than 43° are due to propagating waves (After Kelley et al. 2017 [7], reproduced with permission of the American Geophysical Union).

Figure 2, also from Kelley et al. [7], shows the time interval between STEC enhancements (transitions from blue to red and vice versa, Figure 1). Near simultaneous events are seen between latitude 38°N and 43°N for 140 selected stations indicating that the signals were not travelling at typical ionospheric wave speeds. This demonstrates that the STEC enhancements are tied to the earth and not due to a wave propagating in the ionosphere or upper atmosphere. The sloping points in Figure 2 below latitude 38° N and above latitude 48° N, however, show such propagation in the ionosphere. Such propagating features detected by STEC in GPS data are common phenomenon at mid-latitudes and are also due to electric fields generated in situ in the ionosphere [19].

In this case, the simultaneous GPS signals shown in Figure 2 provide a potential lead time of 40–50 min before the occurrence of the earthquake. Kelley et al. [7], show that the electronic fields generated by vertical displacements of the ionosphere are in response to precursor crustal movements that are capable of generating the simultaneous ionospheric changes.

## 3. Discussion and Conclusions

Substantial evidence exists that animals can detect a range of abiotic sounds. Crustal movements in the earth’s near-surface produce such sounds. The production of this sound, however, is not a direct product of the crustal releases. Instead, a sequence of events are set in motion, starting with crustal failure, occurring prior to an earthquake. These signals are transferred at the speed of light, guided by the earth’s magnetic field, to the ionosphere. Here the crustal signature is detected in the STEC. These STEC fluctuations of the ionosphere are transferred to the earth’s surface and are detectable by ground-based GPS. At this point, they are not in the form of sound waves but trigger vibrations in metal, glass and other surfaces. This latter process, known as electrophonics, generates audible sounds with frequencies ranging between 20 Hz and 20,000 Hz. These are considered to be the sounds that many animals respond to.

The lead time between the precursor crustal movements and the earthquake is indeterminate, but can probably range from tens of minutes to many hours. The ultimate signal, however, represents definitive evidence of the imminent earthquake, an achievement long sought by humans.

The sequence of events described above provides both a testable framework for detecting a precursor earthquake signal and a basis for understanding the inconsistencies observed in animal behavior prior to an earthquake. It is also likely that the severity of the earthquake, magnitude of the audible response and the behavioral reaction of animals are all related. The understanding of this process coupled with the technological ability (GPS), to monitor the magnitude and occurrence of the signals should provide the basis for earthquake prediction. Reliance upon and the use of animal responses to the potential earthquake can thus be refined and improved. Reliable response to the adherent animal behavior could still prove useful, particularly in poorly monitored remote areas.

Methodology which relates the precursor crustal movements to the intensity of the earthquake remains uncertain. Nevertheless, we present a plausible predictive approach which can be subjected to both post and future analysis. Issues relating to how predictions of earthquakes are used go beyond the scope of scientific inquiry.
